# Angelica polysaccharide attenuates LPS-induced inflammation response of primary dairy cow claw dermal cells via NF-κB and MAPK signaling pathways

**DOI:** 10.1186/s12917-021-02952-4

**Published:** 2021-07-19

**Authors:** Mengyue Tian, Ke Li, Ruonan Liu, Jinliang Du, Dongmin Zou, Yuzhong Ma

**Affiliations:** 1 College of Veterinary Medicine, Hebei Agricultural University, 2596 Lekai South Street, Hebei 071001 Baoding, China; 2 International Joint Research Laboratory for Fish Immunopharmacology, Freshwater Fisheries Research Center, Chinese Academy of Fishery Sciences, 214081 Wuxi, Jiangsu China; 3 College of Veterinary Medicine, Shanxi Agricultural University, Shanxi 030801 Taigu, China

**Keywords:** Dairy cow, Claw dermal cell, LPS, Angelica polysaccharide, NF-κB, MAPK

## Abstract

**Background:**

Laminitis, an inflammation of the claw laminae, is one of the major causes of bovine lameness, which can lead to enormous economic losses and animal welfare problems in dairy farms. Angelica polysaccharide (AP) is proved to possess anti-inflammatory properties. But the role of AP on inflammatory response of the claw dermal cells has not been reported. The aim of this study was to investigate the anti-inflammatory effects of AP on lipopolysaccharide (LPS)-induced primary claw dermal cells of dairy cow and clarify the potential mechanisms. In the current research, the primary claw dermal cells were exposed to gradient concentrations of AP (10, 50, 100 µg/mL) in the presence of 10 µg/mL LPS. The levels of cytokines and nitric oxide (NO) were detected with ELISA and Griess colorimetric method. The mRNA expressions of TLR4, MyD88 and chemokines were measured with qPCR. The activation of NF-κB and MAPK signaling pathways was detected with western blotting.

**Results:**

The results indicated that AP reduced the production of inflammatory mediators (TNF-α, IL-1β, IL-6 and NO), downregulated the mRNA expression of TLR4, MyD88 and some pro-inflammatory chemokines (CCL2, CCL20, CXCL2, CXCL8, CXCL10), and suppressed the NF-κB and MAPK signaling pathways evidenced by inhibition of the phosphorylation of IκBα, p65 and ERK, JNK, p38.

**Conclusions:**

Our results demonstrated that AP may exert its anti-inflammatory effects on claw dermal cells of dairy cow by regulating the NF-κB and MAPK signaling pathways.

**Supplementary Information:**

The online version contains supplementary material available at 10.1186/s12917-021-02952-4.

## Background

Lameness is one of the most important and intractable diseases in dairy farms worldwide, which can lead to economic losses and animal welfare problems [1, 2]. Laminitis, a crucial reason for lameness, is defined as diffuse, aseptic, serous inflammation of the corium [3]. It has been estimated that the prevalence of subclinical laminitis in large scale dairy farms was 42.0 % in Thailand [4]. Laminitis can lead to other claw diseases, such as sole hemorrhage, sole ulcer, and white line disease, and is also responsible for milk yield reduction, reproduction disorders and weight loss [5, 6].

The pathogenesis of laminitis has not been fully elucidated. It is currently believed that nutrition disorder is one of the main causes [7]. The disproportion between a high share of rapidly-fermented carbohydrates and low physical effective NDF may cause impaired ruminal health through variation in the VFA concentrations and decreased ruminal fluid pH, which leads to subacute ruminal acidosis (SARA) [8]. In the process, endotoxin, also called lipopolysaccharide (LPS), is released as a result of the massive proliferation and breakdown of bacteria, and then triggers the release of histamine [9]. The accumulation of histamine delays the regeneration of epithelial cells, increases the permeability of rumen wall, which allows the metabolites such as endotoxin to enter the peripheral blood circulation [10]. Endotoxins activate the inflammatory response in claw lamellar tissue, increase the permeability of the capillary wall, lead to the microcirculation disorder of the claw, and ultimately contribute to the appearance of laminitis [11].

At present, there is still no effective method for the prevention and treatment of laminitis. Studies have shown that rumen modifiers like monensin can alleviate the severity of laminitis [7, 12]. However, there are growing concerns about food safety and drug resistance. On the other hand, medicinal plants, due to their low drug resistance and multi-target therapeutic properties, have become a potential source for feed supplements or alternative drugs for various diseases [13].

*Angelica sinensis*, the dried root of *Angelica sinensis*, is a traditional Chinese herbal medicine that has long been used for nourishing, replenishing blood and relieving pain [14]. It has shown that the extracts of *Angelica sinensis* have certain therapeutic effect on blood deficiency model and anemia model in mice [15, 16]. Modern pharmacological studies have demonstrated that *Angelica sinensis* and its extracts possess multiple beneficial properties such as anti-inflammation, antioxidation, antitumor, neuroprotection [17–19]. Angelica polysaccharide (AP), a β-d-pyranoid polysaccharide, is the major bioactive component of *Angelica sinensis* [20]. Accumulating pieces of evidence have confirmed the anti-inflammatory effect of AP. For example, AP mitigated the inflammatory injury induced by LPS in PC-12 cell line [21] and neuronal cell line HT22 [22]. Hence, we hypothesized that AP might have some protective effects on bovine laminitis, but the related literature is limited.

The aim of this study was to investigate the potential protective effects of AP on LPS-induced inflammatory claw dermal cells *in vitro*, and provide theoretical basis for the application of AP on bovine laminitis in the future.

## Results

### Cytotoxicity of AP on claw dermal cells

**Fig. 1:**
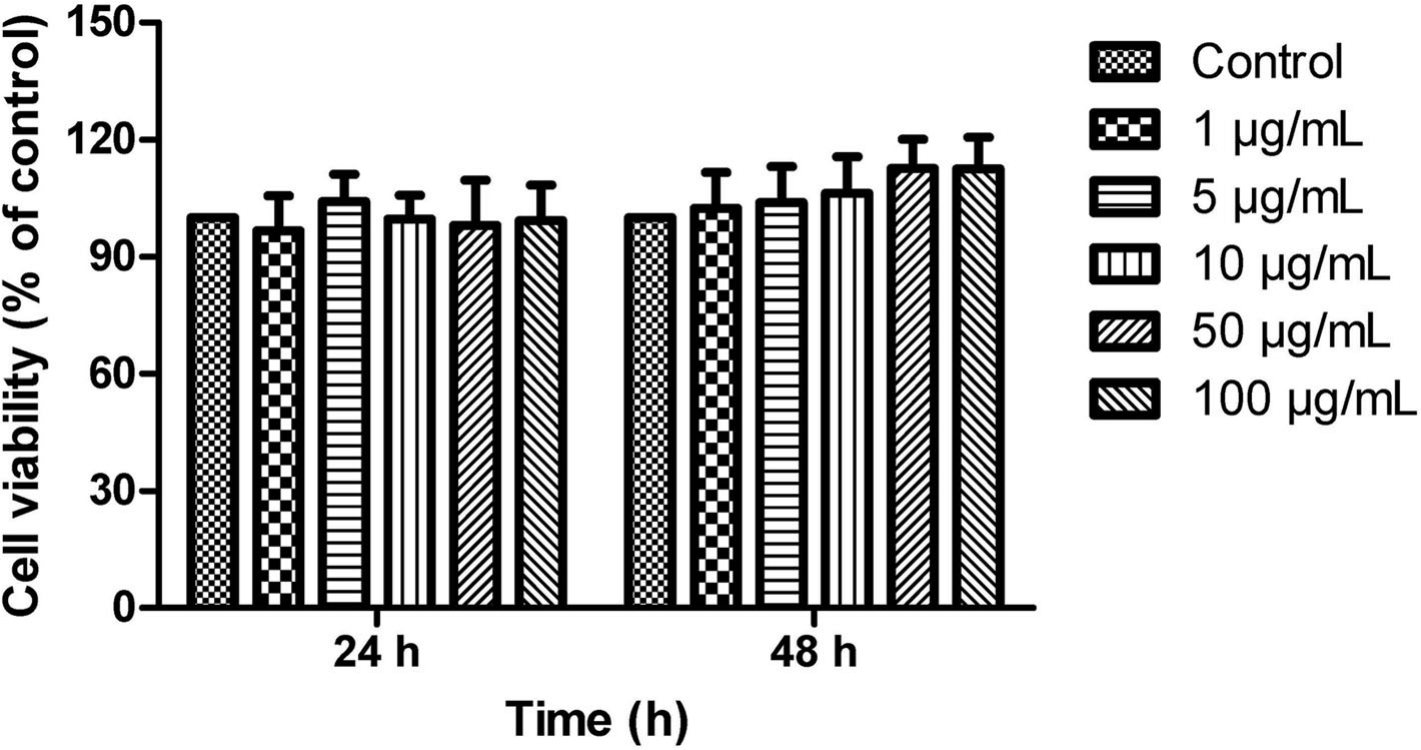
Cytotoxic of angelica polysaccharide (AP) on claw dermal cells. Cell viability was measured by cell counting kit-8 (CCK-8) method with the treatment of various concentrations (1, 5, 10, 50, 100 µg/mL) of AP for 24 or 48 h. The data were presented as mean ± standard deviations (*n* = 6)

As shown in Fig. 1, the indicated concentrations of AP treated for 24 or 48 h had no cytotoxic effects on claw dermal cells. Thus, 1, 5, 10, 50, 100 µg/mL AP could be used for further research.

### Effects of AP on the levels of inflammatory mediators

**Fig. 2:**
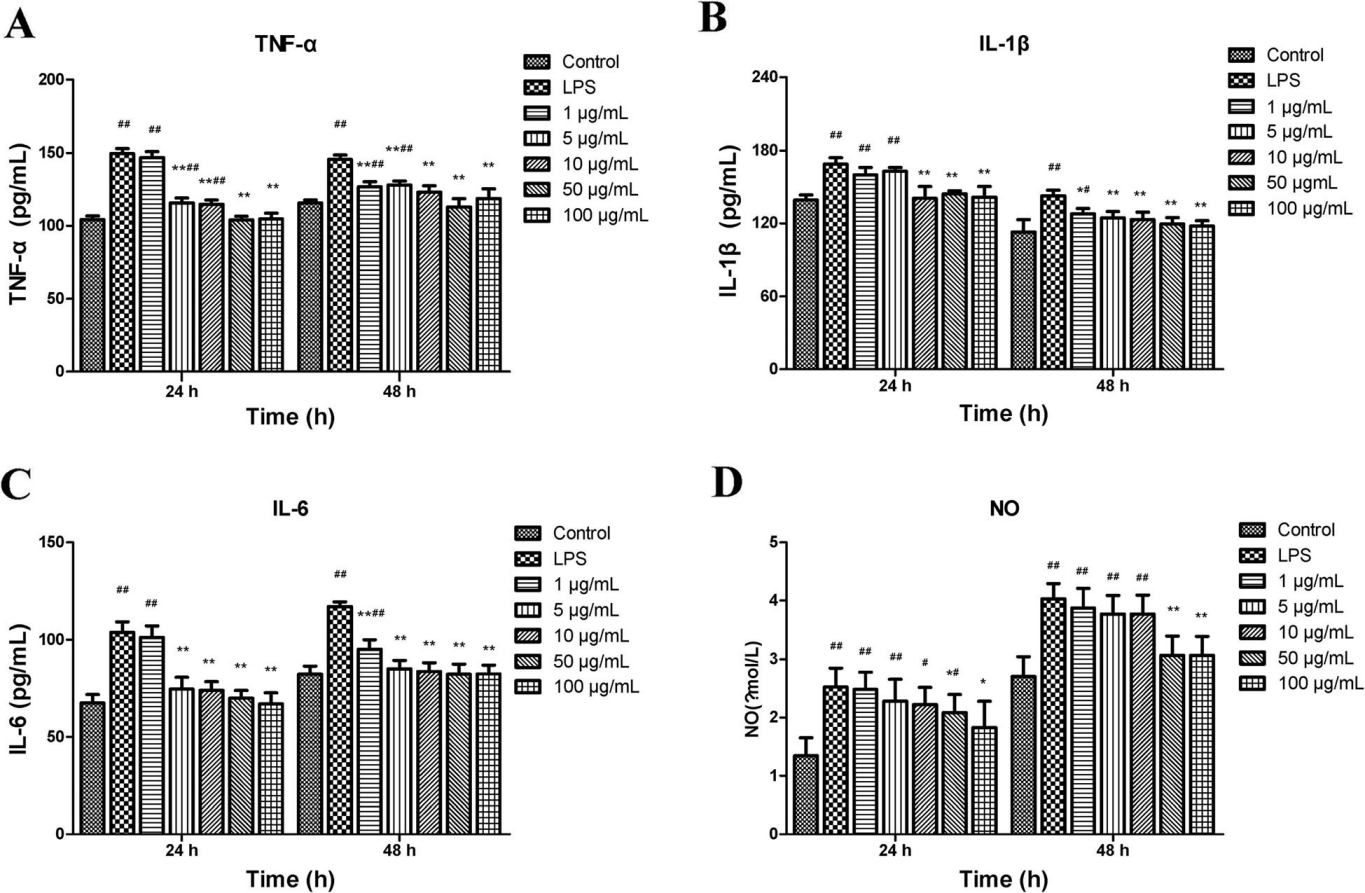
Effects of angelica polysaccharide (AP) on the levels of tumor necrosis factor-α (TNF-α) (**A**), interleukin-1β (IL-1β) (**B**), interleukin-6 (IL-6) (**C**), nitric oxide (NO) (**D**) in LPS-induced claw dermal cells. Cells were exposed to various concentrations of AP (1, 5, 10, 50, 100 µg/mL) with or without the presence of 10 µg/mL LPS for 24 and 48 h. The data were presented as mean ± standard deviations (*n* = 6). *P < 0.05 vs. LPS model group; **P < 0.01 vs. LPS model group. ^#^*P* < 0.05 vs. control group; ^##^*P* < 0.01 vs. control group

LPS induced an increase in the levels of TNF-α, IL-1β, IL-6, NO compared with the control group (*P* < 0.01) (Fig. 2).

Administration of 1 µg/mL AP had no difference in TNF-α and IL-6 levels compared with those in the LPS-treated group for 24 h (*P* > 0.05), but could significantly reduce the levels of TNF-α and IL-6 at 48 h. The other doses of AP significantly reduced the levels of TNF-α and IL-6 for the indicated times (*P* < 0.01) (Fig. 2A,C).

With 1, 5 µg/mL AP treatment for 24 h, there was no significant difference of IL-1β level compared with that in the LPS-treated group (*P* > 0.05), while 10, 50, 100 µg/mL AP significantly reduced the IL-1β level (*P* < 0.01). The indicated doses of AP obviously reduced the IL-1β level at 48 h (Fig. 2B).

Treatment with 50, 100 µg/mL AP reduced the NO level at 24 h (*P* < 0.05) and at 48 h (*P* < 0.01), while no significant difference was found in NO compared to LPS-treated group (*P* > 0.05) using other doses (Fig. 2D).

Based on the above results, 10, 50, 100 µg/mL AP performed better anti-inflammatory effects. Therefore, the concentrations of 10, 50, 100 µg/mL AP were used for further research.

### Effects of AP on relative mRNA levels of chemokines

**Fig. 3:**
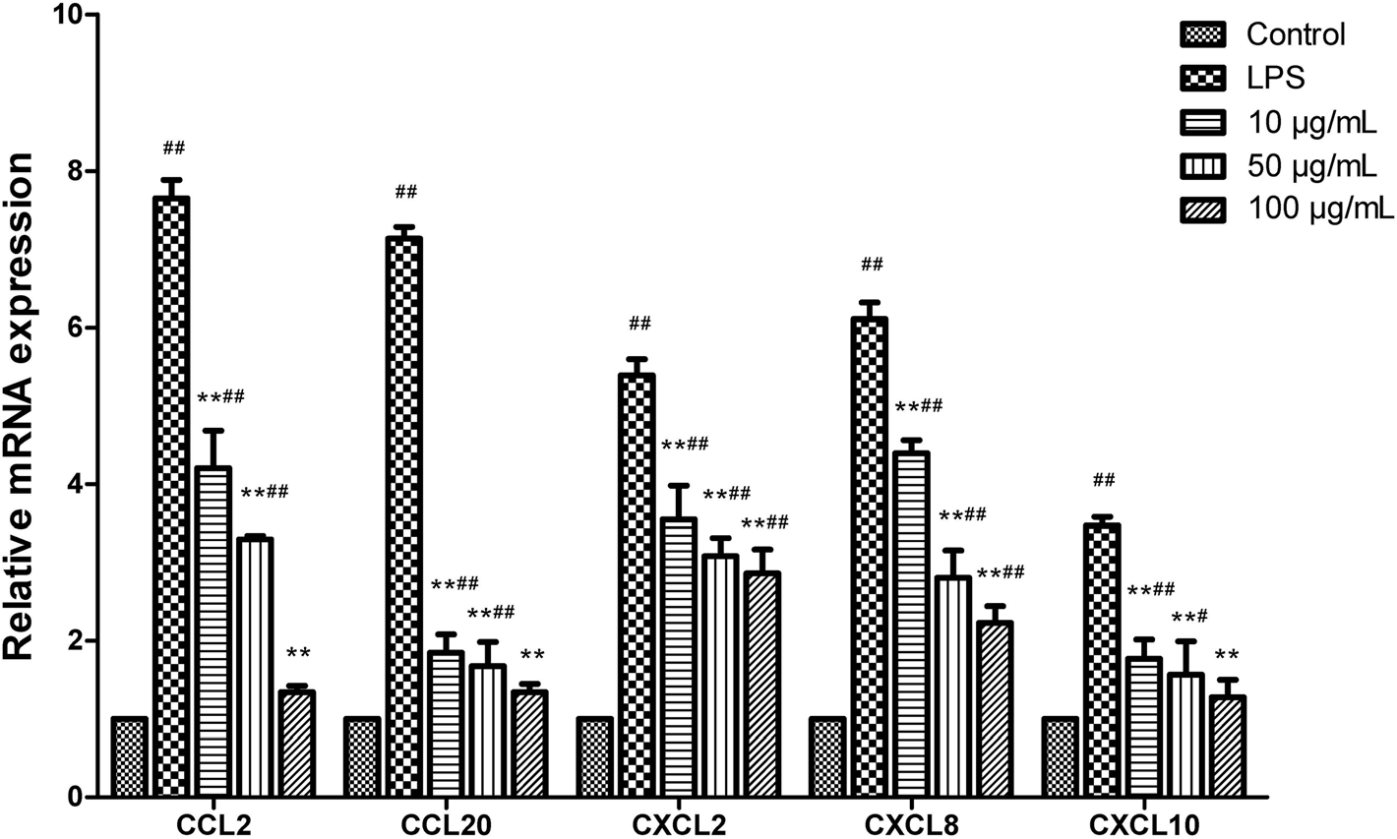
Effects of angelica polysaccharide (AP) on mRNA expression levels of chemokines (CCL2, CCL20, CXCL2, CXCL8, CXCL10) in LPS-induced claw dermal cells. Cells were exposed to various concentrations of AP (10, 50, 100 μg/mL) with or without the presence of 10 μg/mL LPS for 48 h. The data were presented as mean ± standard deviations (*n* = 6). *P< 0.05 vs. LPS model group; **P < 0.01 vs. LPS model group.^#^*P* < 0.05 vs. control group;^##^*P* < 0.01 vs. control group

As shown in Fig. 3, the gene expression levels of CCL2, CCL20, CXCL2, CXCL8, CXCL10 were upregulated significantly in response to LPS challenge. AP treatment downregulated significantly the gene expression levels of these chemokines compared with those in LPS-treated group (*P* < 0.01) in a dose dependent manner.

### Effects of AP on relative mRNA levels of TLR4 and MyD88

**Fig. 4:**
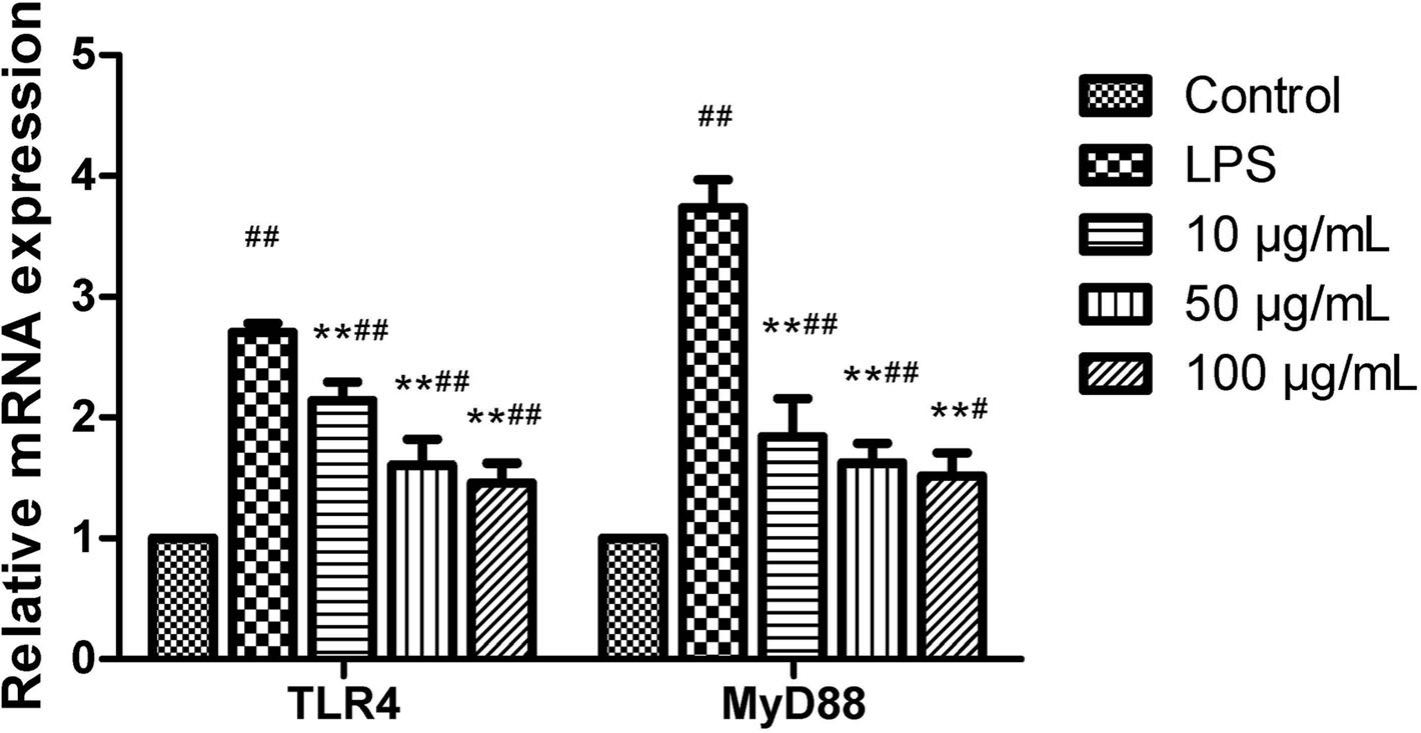
Effects of angelica polysaccharide (AP) on mRNA expression levels of toll-like receptor 4 (TLR4) and myeloid differentiation factor 88 (MyD88) in LPS-induced claw dermal cells. Cells were exposed to various concentrations of AP (10, 50, 100 μg/mL) with or without the presence of 10 μg/mL LPS for 48 h. The data were presented as mean ± standard deviations (*n* = 6). *P < 0.05 vs. LPS model group; **P < 0.01 vs. LPS model group.^#^*P*< 0.05 vs. control group;^##^*P* < 0.01 vs. control group

As shown in Fig. 4, LPS upregulated markedly the mRNA levels of TLR4 and MyD88 compared with those in the control group (*P* < 0.01). All dosages of AP treatment suppressed significantly these gene expression levels at the indicated times (*P* < 0.01).

### Effects of AP on protein expression of NF-κB and MAPKs pathways

**Fig. 5:**
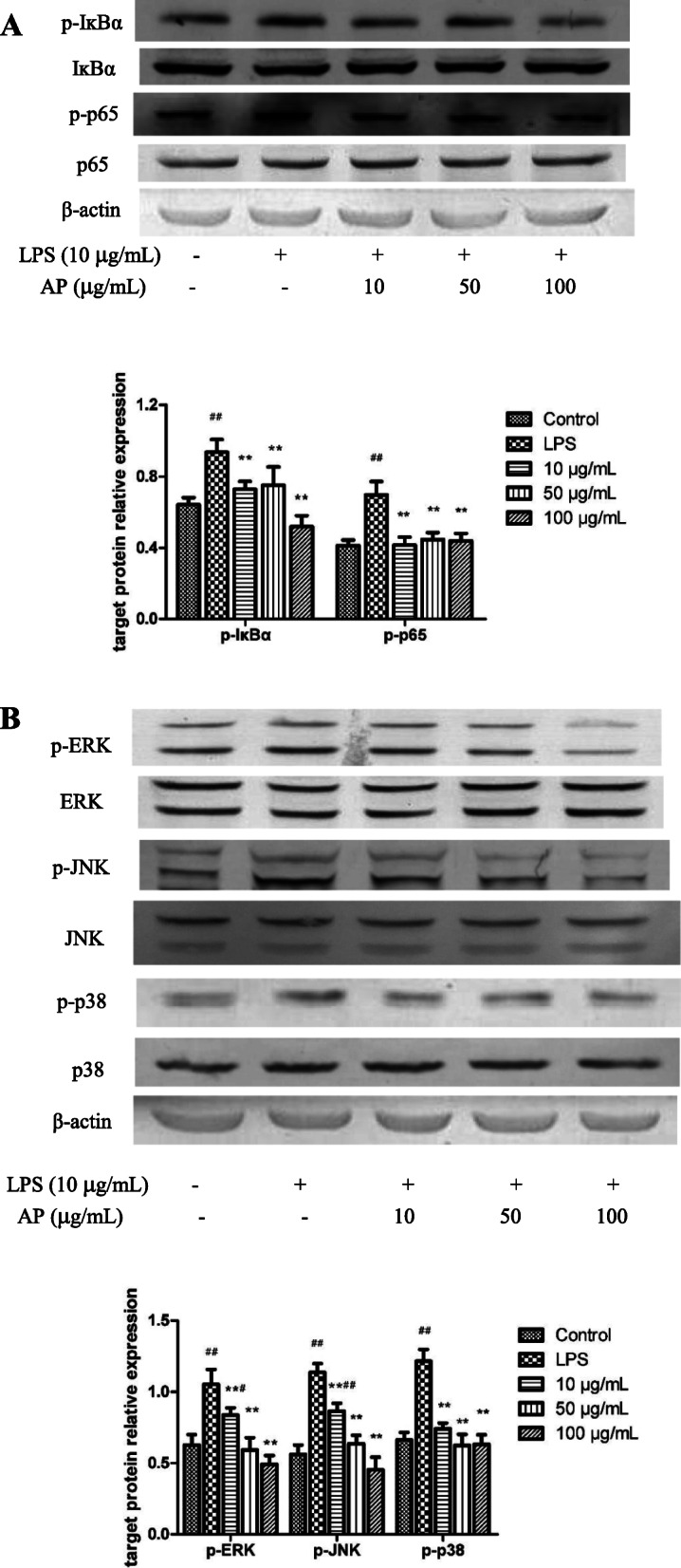
Effects of angelica polysaccharide (AP) on protein expression of nuclear factor-κB (NF-κB) (**A**) and mitogen-activated protein kinase (MAPK) (**B**) signaling pathways in LPS-induced claw dermal cells. Cells were exposed to various concentrations of AP (10, 50, 100 µg/mL) with or without the presence of 10 µg/mL LPS for 48 h. The data were presented as mean ± standard deviations (*n* = 6). *P < 0.05 vs. LPS model group; **P < 0.01 vs. LPS model group. ^#^*P* < 0.05 vs. control group; ^##^*P* < 0.01 vs. control group

It was shown in Fig. 5 that LPS increased significantly the protein levels of p-p65, p-IκBα, p-ERK, p-JNK, p-p38 compared with the control group (*P* < 0.01). In comparison with the LPS-treated group, 10, 50, 100 µg/mL AP treated for 48 h reduced the protein levels of p-p65, p-IκBα, p-ERK, p-JNK, p-p38 significantly (*P* < 0.01).

## Discussion

Laminitis, an inflammation of the claw laminae, is one of the most significant diseases in dairy industry, accounting for 41 % cases of bovine lameness [3]. The laminitis-related diseases not only lead to low milk production, but also relate to weight loss and reproductive capacity reduction, resulting in hidden costs of dairy farming [23].

Lipopolysaccharide (LPS), also known as endotoxins, plays a crucial role in the pathologies of laminitis. Elevated LPS concentrations in plasma were detected in both subclinical and chronic laminitis dairy cows [24], as well as in the high-grain diet model of goats [25]. Lipopolysaccharide transported to the peripheral blood could activate the inflammatory response in the lamellar tissue and result in laminar damage [26]. Additionally, LPS with higher concentrations had negative effects on the tissue integrity of hoof explants *in vitro* [27]. In our previous study, an inflammatory model on claw dermal cells with 10 µg/mL LPS had established [28]. Therefore, in this study, LPS was used as a stimulus to establish an inflammatory model of claw dermal cells to explore the possible therapeutic drug for laminitis *in vitro*.

The anti-inflammatory effects of AP had been verified in previous studies [29, 30], but the effects on claw dermal cells remain unclear. It is widely accepted that the excessive production of some inflammatory mediators and chemokines are tightly related to the inflammatory injury [31]. TNF-α, IL-1β and IL-6 are typical pro-inflammatory cytokines that play a pivotal role in the pathogenesis of inflammatory response [32]. Previous studies have reported that the IL-1β and IL-6 expression in laminar was implicated in the degree of lamellar injury [33]. The higher levels of TNF-α and IL-6 in plasma were also detected in laminitis cows [24]. In addition, the secretion of these cytokines can affect the synthesis of mediators such as PG, leukotriene, and NO [34]. As an intracellular messenger molecule, NO regulates the various functions of cells and involves in the defense functions of the immune system [35]. Excessive secretion of NO leads to cytotoxicity and mediates the inflammation progress [36]. Chemokines, or chemotactic cytokines, are small heparin-binding proteins that direct the migration and positioning of immune cells, and play a crucial role in the inflammation injury and innate immune system [37, 38]. The CCL2, CCL20, CXCL2, CXCL8, and CXCL10 measured in this experiment are common pro-inflammatory chemokines from the CCL and CXCL subfamily of chemokines, which actively participate in the inflammatory response under some pro-inflammatory stimuli like LPS, IL-1, TNF-α [39]. Together, these pro-inflammatory substances could exhibit cytotoxic effects and accelerate the inflammatory response [40]. The reduction of these inflammatory indicators could serve as a therapy signal for inflammation amelioration.

Our results showed that AP markedly reduced the production of TNF-α, IL-1β, IL-6, and NO, and downregulated the mRNA expression of CCL2, CCL20, CXCL2, CXCL8, CXCL10, indicating that AP alleviated the LPS-induced inflammatory response in claw dermal cells by inhibiting the production of inflammatory mediators and chemokines.

It is widely known that toll-like receptor 4 (TLR4) is the main receptor for LPS stimulation [41]. Once recognized, the LPS complex is capable of initiating a series of cascades via the stimulation of myeloid differentiation factor 88 (MyD88), including the initiation of NF-κB and MAPK signaling pathways, and ultimately induces inflammatory cytokine and chemokine expression [42].

Nuclear factor-κB (NF-κB) is a transcription factor, which involves in the regulation of multiple cell functions, such as proliferation, apoptosis and inflammation, and plays a crucial role in the regulation of pro-inflammatory genes [43]. The predominant form of NF-κB is heterodimer p50-p65 [44]. In an unstimulated state, NF-κB is located in cytoplasm under a silent state because of the binding with κB inhibitor (IκB). Stimulator like LPS could induce the phosphorylation and degradation of IκBα, followed by NF-κB/IκB complex dissociation, as well as the NF-κB phosphorylation. Then, the phosphorylated NF-κB translocated into the nucleus and induced the expression of pro-inflammatory mediators [45].

Mitogen-activated protein kinases (MAPKs) are a family of serine/ threonine protein kinases, consisting of extracellular signal-regulated kinase 1/2 (ERK1/2), p38, and c-Jun NH2-terminal kinase (JNK) [46]. The MAPK signaling pathway plays an important role in the early stage inflammatory responses and the activation of NF-κB pathway [47]. Previous studies have demonstrated that AP exerted its anti-inflammatory effect through repression of NF-κB and MAPK pathways [22, 48]. Our data showed that AP restrained the initiation of NF-κB and MAPK pathways on claw dermal cells by decreasing the phosphorylation of IκBα, p65 and ERK, JNK, p38, which is consistent with the previous studies.

## Conclusions

In conclusion, the present study demonstrated that AP protects the primary claw dermal cells of dairy cow against LPS-induced inflammatory injury by decreasing the pro-inflammatory mediators and chemokines, which may be regulated by NF-κB and MAPK signaling pathways. Altogether, AP might serve as a suitable therapeutic candidate for the management of bovine laminitis, the biological significance of these results needs to be investigated further in the larger *in vivo* field trials.

## Methods

### Materials

Dulbecco’s Modified Eagle Medium (DMEM), fetal bovine serum (FBS) and 1×Insulin-Transferrin-Selenium (ITS) were obtained from Gibco (Grand Island, NY). LPS (*Escherichia coli* O55: B5) was purchased from Sigma (St. Louis, USA). Angelica polysaccharide (≥ 90 % purity), cell counting kit-8 (CCK-8), RIPA cell lysis buffer, BCA protein assay kit, and NBT/BCIP chromogen kit were acquired from Solarbio (Beijing, China). ELISA kits of tumor necrosis factor-α (TNF-α) (DG90837Q), interleukin (IL)-1β (DG90995Q) and IL-6 (DG90838Q) for dairy cow were purchased from DG Biotech Co. Ltd. (Beijing, China). Nitric oxide (NO) kit (A013-2-1) was purchased from Nanjing Jiancheng Bioengineering Institute (Nanjing, China). The primary antibodies against phosphor-JNK, phosphor-ERK, phosphor-p38, JNK, ERK, and p38 were obtained from Cell Signaling Technology (Danvers, MA, USA). Antibodies for phosphor-IκBα, phosphor-p65 NF-κB, IκBα, p65 NF-κB and β-actin were obtained from Bioss (Woburn, MA, USA). The HRP-conjugated secondary antibody was purchased from ZSGB-Bio (Beijing, China). Ultrapure RNA extraction kit and HiFiscript cDNA Synthesis Kit were purchased from CWBIO (Beijing, China). 2×Fast Super EvaGreen® qPCR Mastermix was acquired from US Everbright Inc.(CA, USA). All reagents used in this study were pyrogen-free (Horseshoe Crab Reagent Manufactory, Xiamen, China). All other chemicals were of reagent grade.

### Cell culture and treatment

Claw dermal cells of dairy cow were isolated using the tissue adherent culture method as previously described [28]. Claw lamellar tissues were collected at a local abattoir from adult healthy dairy cows. The tissues were aseptically cut off and put into sterile saline solution with antibiotics (200 units/mL of *Penicillin*, 200 µg/mL of *Streptomycin*), and then transported on ice to the laboratory within 2 h. The acquired tissues were washed with sterile phosphate buffered saline (PBS) for 3 times, trimmed into small pieces and soaked in 0.25 % trypsin solution at 4℃ for 18–24 h. After rinsing with PBS, the epidermis and dermis were separated. The dermis pieces were seeded onto 6-well plates coated with rat tail collagen, cultured in DMEM, supplemented with 15 % FBS, 1×ITS, 0.025 M HEPES, 200 units/mL of *Penicillin*, 200 µg/mL of *Streptomycin*, and maintained in 5 % CO_2_ incubator at 37℃. Medium was replaced every 2–3 days. Tissue pieces were removed when cells were about 50 % confluence. Upon 80–90 % confluence, cells were detached with 0.25 % trypsin-EDTA solution and seeded into 25 cm^2^ flasks.

The claw dermal cells were exposed to different concentrations of AP (1, 5, 10, 50, 100 µg/mL) with or without the stimulation of 10 µg/mL LPS for different times based on different experimental conditions.

### Cell viability assay

Cell viability was measured using the Cell Counting Kit-8 (CCK-8). Claw dermal cells were seeded into 96-well plates at a density of 1 × 10^5^ cells/ well and cultured until 80–90 % confluency. The cells were treated with different concentrations of AP (1-100 µg/mL) for 24 and 48 h. Then 10 µL CCK-8 was added into each well. The cells were incubated at 37℃ for 1 h. The absorbance at 450 nm was measured by a microplate reader (Bio-Rad, CA, USA).

### Cytokine measurement

The levels of TNF-α, IL-1β and IL-6 in supernatants of claw dermal cells were detected using commercial ELISA kits, according to the manufacturer’s guidelines. The OD value at a wavelength of 450 nm was measured by a microplate reader (Bio-Rad, CA, USA). The NO concentration in dermal cell supernatant was measured using Griess colorimetric method, following the manufacturer’s protocol. Absorbance was measured at 550 nm by a microplate reader (Bio-Rad, CA, USA). Draw a standard curve with the standard solution concentrations as the horizontal axis and the measured OD value as the vertical axis, then calculate the concentrations of cytokines on the basis of standard curve. The intra- and inter- CV (coefficients of variations) were less than 15 %.

### Quantitative real-time PCR analysis

Total RNA was extracted from dermal cells using Ultrapure RNA extraction kit following the manufacturer’s protocol. The concentration and purity (OD260/OD280 absorption ratio > 1.8) of total RNA were evaluated by a NanoDrop 2000 spectrophotometer (Thermo Scientific, Ottawa, ON, Canada). Subsequently, the total RNA was reverse transcribed into cDNA with the use of HiFiscript cDNA Synthesis Kit according to the manufacturer’s instructions. Quantitative real-time PCR was performed using 2× Fast Super EvaGreen® qPCR Mastermix on a LightCycler96 Real-Time PCR system (Roche, Basel, Switzerland). Primer sequences are listed in Table 1. The following cycling conditions were performed: 95 °C for 300 s, 40 cycles of 95 °C for 5 s, 56 °C for 30 s and 72 °C for 15 s. Negative control sample with water was set in the reaction mixture instead of cDNA templates. The relative expression of target genes was normalized relative to the level of the control (GAPDH) and calculated using the 2^−ΔΔCt^ method [49].

**Table 1:** Primer sequences used for amplification of qPCR

Accession No.	Genes^a^	Sense	Antisense	Product size(bp)
NM_174198.6	TLR4	AGCTTCAACCGTATCATGGCCTCT	ACTAAGCACTGGCATGTCCTCCAT	166
NM_001014382.2	MyD88	AAGTACAAGCCAATGAAGAAAGAG	GAGGCGAGTCCAGAACCAG	102
NM_174006.2	CCL2	CGCTCAGCCAGATGCAATTA	GACCCATTTCTGCTTGGGGT	184
NM_174263.2	CCL20	TTGATGTCAGTGCTATTGCT	ACCCACTTCTTCTTTGGATC	209
NM_174299.3	CXCL2	ACCGAAGTCATAGCCACTCTC	TCCAGATGGCCTTAGGAGGT	218
NM_173925.2	CXCL8	AAACACATTCCACACCTTTC	TCTTCACAAATACCTGCACA	171
NM_001046551	CXCL10	CTCGAACACGGAAAGAGGCA	TCCACGGACAATTAGGGCTT	117
NM_001034034.2	GAPDH	CACCCTCAAGATTGTCAGCA	GGTCATAAGTCCCTCCACGA	103

^a^TLR4, toll-like receptor 4, MyD88, myeloid differentiation factor 88, *GAPDH, *Glyceraldehyde 3-phosphate dehydrogenase

### Western blot analysis

Total proteins from claw dermal cells were extracted with RIPA cell lysis buffer and quantified by BCA protein assay kit. Total protein (20–50 µg/sample) was separated on 12 % SDS-polyacrylamide gel and transferred to nitrocellulose membranes. Nonspecific binding sites of membranes were blocked with 5 % non-fat milk for 1 h at room temperature. Membranes were incubated with antibodies specific for JNK, phosphor-JNK, ERK, phosphor-ERK, p38, phosphor-p38, IκBα, phosphor-IκBα, p65 NF-κB, phosphor-p65 NF-κB and β-actin at 4 °C overnight, and then incubated with HRP-conjugated secondary antibodies at room temperature for 1 h. Immunoblot signals were visualized with NBT/BCIP chromogen kit. Densitometric values were obtained from 3 separate experiments using ImageJ software (NIH, Bethesda, MD).

### Statistical analysis

Data were presented as mean ± standard deviations (SD) of at least three independent experiments. The statistical analyses were performed with GraphPad Prism 5 (GraphPad Software, La Jolla, USA). Significant differences were evaluated by one-way analysis of variance (ANOVA) with Duncan’s post hoc test using SPSS 21.0 software (SPSS Inc., Chicago, IL). *P* < 0.05 was considered as statistically significant.

## Supplementary Information

**Additional file 1.** The original, full length blots of western blot.

## Data Availability

The datasets used and/or analysed during the current study are available from the corresponding author on reasonable request. The original sequences we used for primer sequences design can be found in GenBank under the accession numbers: NM_174198.6, NM_001014382.2, NM_174006.2, NM_174263.2, NM_174299.3, NM_173925.2, NM_001046551, NM_001034034.2.

## Abbreviations

AP: Angelica polysaccharide
CCK-8: Cell counting kit-8
DMEM: Dulbecco’s Modified Eagle Medium
ELISA: Enzyme-linked immunosorbent assay
FBS: Fetal bovine serum
IκBα: κB inhibitor α
IL-1β: Interleukin-1β
ITS: Insulin-Transferrin-Selenium
LPS: Lipopolysaccharides
MAPK: Mitogen-activated protein kinases
MyD88: Myeloid differentiation factor 88
NF-κB: Nuclear factor-κB
NO: Nitric oxide
PBS: Phosphate buffered saline
TNF-α: Tumor necrosis factor-α
TLR4: Toll-like receptor 4
