# Supplementary material for: Angelica polysaccharide attenuates LPS-induced inflammation response of primary dairy cow claw dermal cells via NF-κB and MAPK signaling pathways
Source: BMC Vet Res. 2021 Jul 19;17:248. doi: 10.1186/s12917-021-02952-4 (PMC8287747; doi:10.1186/s12917-021-02952-4)
Supplement: Supplementary file 1 — Additional file 1. The original, full length blots of western blot. [file 12917_2021_2952_MOESM1_ESM.docx]

Additional file 1. The original, full length blots of western blot.

p-IĸBα

LPS (10 μg/mL) - + + + +

AP (μg/mL) - - 10 50 100


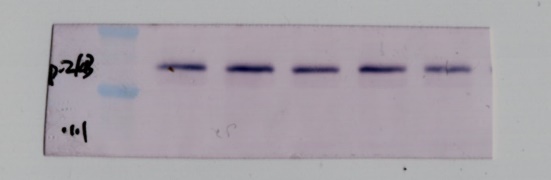


35 kDa

45 kDa

36 kDa

IĸBα


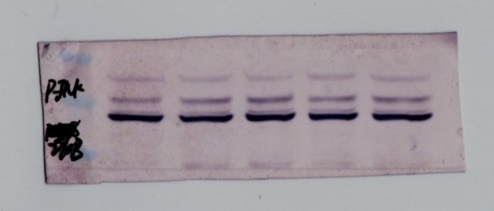


35 kDa

34 kDa

LPS (10 μg/mL) - + + + +

AP (μg/mL) - - 10 50 100

p-p65


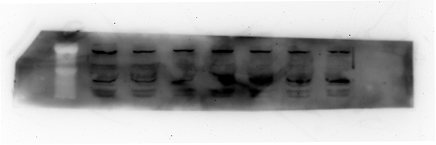


60 kDa

61 kDa

LPS (10 μg/mL) - + + + +

AP (μg/mL) - - 10 50 100

p65


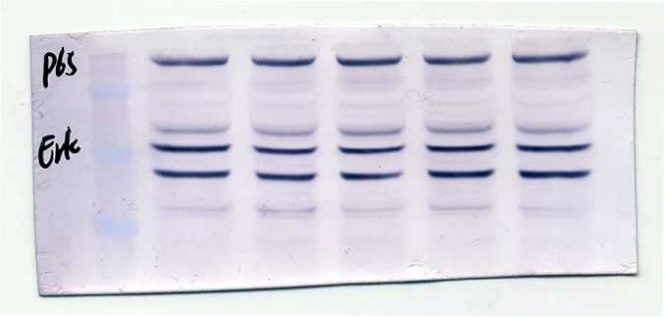


61 kDa

60 kDa

45 kDa

35 kDa

LPS (10 μg/mL) - + + + +

AP (μg/mL) - - 10 50 100


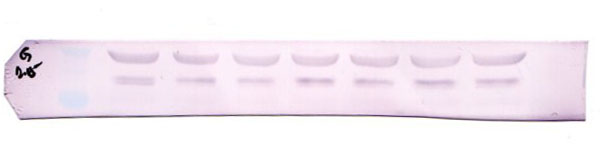


β-actin

45 kDa

42 kDa

35 kDa

LPS (10 μg/mL) - + + + +

AP (μg/mL) - - 10 50 100


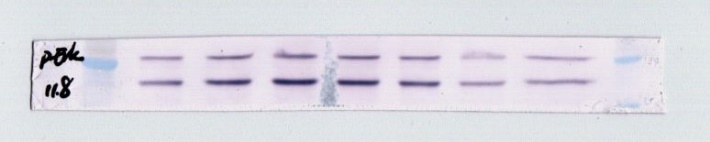


p-ERK

45 kDa

44 kDa

LPS (10 μg/mL) - + + + +

AP (μg/mL) - - 10 50 100

42 kDa

ERK


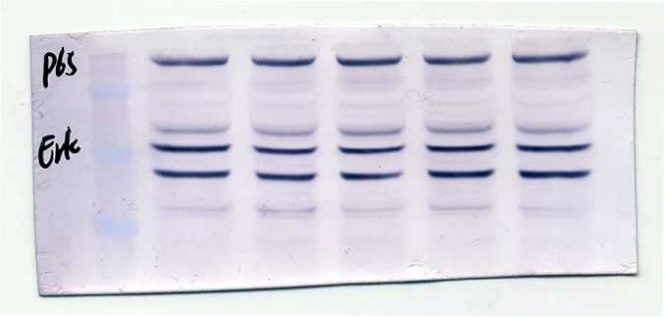


44 kDa

60 kDa

45 kDa

35 kDa

LPS (10 μg/mL) - + + + +

AP (μg/mL) - - 10 50 100

42 kDa


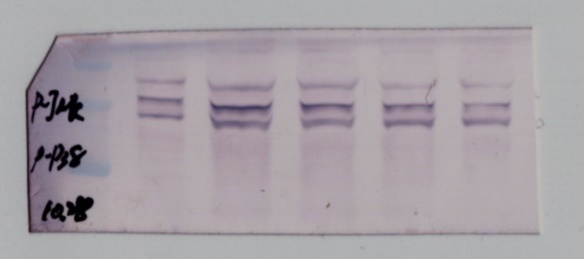


p-JNK

54 kDa

60 kDa

45 kDa

LPS (10 μg/mL) - + + + +

AP (μg/mL) - - 10 50 100

46 kDa


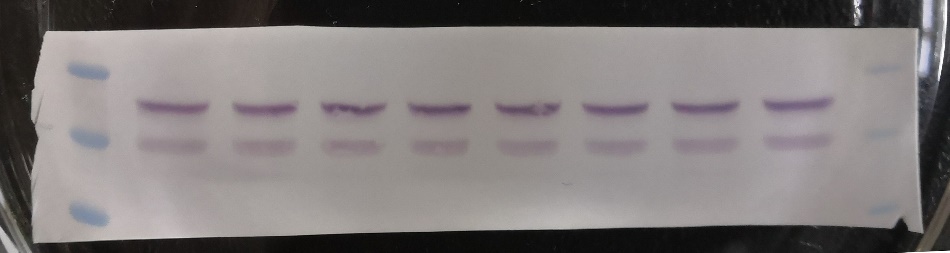


JNK

54 kDa

60 kDa

45 kDa

LPS (10 μg/mL) - + + + +

AP (μg/mL) - - 10 50 100

46 kDa

p-p38


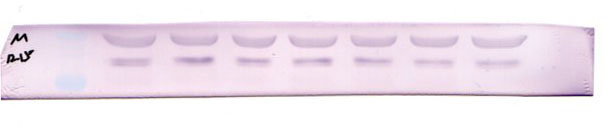


40 kDa

45 kDa

35 kDa

LPS (10 μg/mL) - + + + +

AP (μg/mL) - - 10 50 100


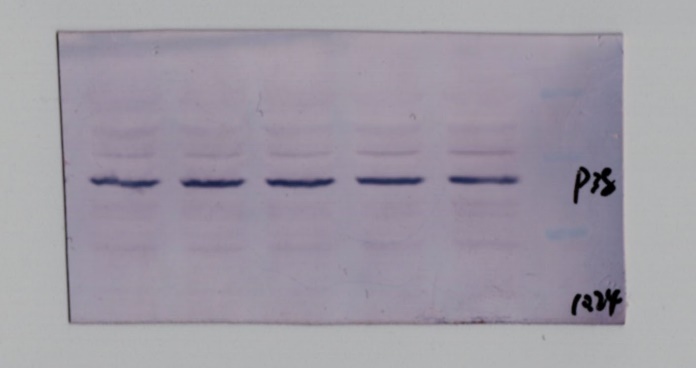


p38

40 kDa

45 kDa

35 kDa

LPS (10 μg/mL) - + + + +

AP (μg/mL) - - 10 50 100

β-actin


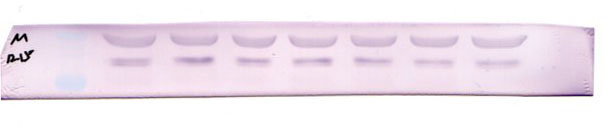


45 kDa

42 kDa

35 kDa

LPS (10 μg/mL) - + + + +

AP (μg/mL) - - 10 50 100
